# Photoelectrochemical Valorization of Plastic Waste Using Catalytic Silicon Photoanodes

**DOI:** 10.1002/cssc.70852

**Published:** 2026-06-30

**Authors:** Manel Machreki, Marielle Blot, Gabriel Loget, Patrick Garrigue, Isabelle Soutrel, Bruno Fabre

**Affiliations:** ^1^ Univ Rennes CNRS ISCR (Institut des Sciences Chimiques de Rennes) – UMR 6226 Rennes France; ^2^ University of Bordeaux Bordeaux INP ISM UMR CNRS 5255 Pessac France; ^3^ Univ Rennes École Nationale Supérieure de Chimie de Rennes CNRS ISCR – UMR 6226 Rennes France

**Keywords:** ethylene glycol oxidation, photoelectrochemistry, plastic upcycling, polyethylene terephthalate, silicon photoanodes

## Abstract

Solar plastic upcycling is demonstrated through the photoelectrochemical oxidation of ethylene glycol derived from real world, pretreated polyethylene terephthalate (PET) bottle waste on an n‐Si/Ni photoanode, producing formic acid and glycolic acid with high Faradaic efficiency. The catalytic silicon photoanode promotes efficient interfacial charge transfer under simulated solar illumination in alkaline media, enabling selective oxidation of PET‐derived ethylene glycol. Mechanistic insights obtained from electrochemical and spectroscopic analyses indicate that nickel oxyhydroxide species facilitate C─C bond cleavage through surface‐bound intermediates. These findings highlight the potential of silicon‐based photoanodes as promising platforms for solar‐driven plastic waste valorization and sustainable carbon recycling.

## Graphical abstract

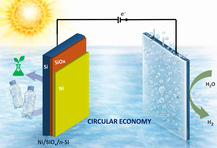

## 1 Introduction

Polyethylene terephthalate (PET), extensively used in disposable beverage bottles, represents one of the largest and most persistent sources of plastic waste worldwide [1]. The durability and chemical stability that make PET attractive for packaging also contribute to its environmental accumulation, motivating the development of sustainable recycling and upcycling strategies. Conventional mechanical recycling often suffers from polymer chain scission, contamination, and loss of material properties, limiting its long‐term viability [2]. In contrast, chemical upcycling aims to convert waste plastics into value‐added chemicals, offering an attracting route to retain or enhance economic value while closing the materials loop. Among chemical recycling strategies, PET depolymerization has attracted significant attention, as it yields well‐defined molecular intermediates, most notably ethylene glycol (EG) [3]. EG is an attractive platform molecule due to its high solubility, low toxicity, and potential to undergo selective oxidation into C_1_ and C_2_ oxygenates such as formic acid (FA) and glycolic acid (GA), which are widely used in chemical, pharmaceutical, and energy‐related applications. However, conventional oxidation routes typically require harsh conditions, elevated temperatures, or stoichiometric oxidants, undermining overall sustainability [4].

Photoelectrochemical (PEC) systems provide an alternative framework by directly coupling solar energy conversion with chemical transformations [5]. In PEC oxidation, photogenerated holes drive substrate oxidation at the photoanode, while avoiding or suppressing the kinetically sluggish oxygen evolution reaction (OER) [6]. This approach enables oxidative valorization reactions to proceed under ambient conditions with improved energy efficiency. Recent studies have extended PEC oxidation to PET and PET‐derived intermediates, but understanding and steering C─C bond scission selectivity is still at an early stage [7].

Silicon‐based photoanodes are particularly attractive for PEC oxidation owing to their suitable bandgap, strong visible‐light absorption, earth abundance, and technological maturity. Surface modification with transition metal catalysts, such as nickel, can further enhance interfacial charge transfer and catalytic selectivity while effectively protecting the semiconductor substrate against deleterious reactions (oxidative degradation and corrosion) [8]. Under anodic bias and alkaline conditions, nickel surfaces are converted to nickel oxyhydroxide (NiOOH) species, which are widely recognized as active phases for alcohol and urea oxidation [9, 10]. These NiOOH species mediate oxidation through surface‐bound intermediates, enabling C─C bond cleavage pathways while improving charge utilization.

Ni‐based cocatalysts have emerged as important redox‐active interfaces for PEC PET oxidation, as demonstrated in Fe_2_O_3_/Ni(OH)_*x*_, Ti–Fe_2_O_3_/Ni(OH)_*x*_, Ni phosphate/α‐Fe_2_O_3_, BiVO_4_/NiCo‐LDH (LDH = layered double hydroxides), and TiO_2_–Ni(OH)_2_‐based photoanodes [11, 15]. These studies established that Ni^2+^/Ni^3+^ redox cycling can efficiently mediate the oxidation of PET‐derived EG and promote selective C─C bond cleavage toward FA. However, most reported systems rely on oxide semiconductor photoabsorbers, and, to the best of our knowledge, silicon‐based MIS photoanodes have not yet been explored for PEC PET plastic waste oxidation. The originality of the present work lies in applying a crystalline silicon‐based MIS photoanode to PEC PET plastic waste oxidation. In this Ni/SiO_*x*_/*n*‐Si architecture, the thin Ni layer is integrated into the photoanode structure as a multifunctional catalytic contact rather than being used only as a surface cocatalyst.

Here, we demonstrate for the first time that an Ni/SiO_*x*_/*n*‐Si photoanode efficiently achieves solar‐driven PEC oxidation of PET‐bottle hydrolysate (PET_HLS_), selectively converting EG into FA and GA. This work establishes a mechanistically guided route for PET bottle upcycling through PEC oxidation and highlights the role of nickel oxyhydroxide species in steering selective plastic‐derived C─C bond transformations.

## 2 Results and Discussion

Ni/SiO_*x*_/Si metal–insulator–semiconductor (MIS) photoanodes were fabricated by first chemically oxidizing Si (100) wafers to form a 1.5 nm‐thick SiO_*x*_ tunnel layer, followed by DC magnetron sputtering of Ni films with thicknesses of 20 and 60 nm, as described in our previous work [8, 9]. The resulting photoanodes were further characterized by SEM, EDS, AFM, and X‐ray photoelectron spectroscopy (XPS) to evaluate the morphology, thickness, surface roughness, and elemental composition of the Ni/SiO_*x*_/*n*‐Si architecture. Surface and cross‐sectional SEM images confirmed the formation of homogeneous and continuous Ni coatings, with thicknesses close to the nominal sputtered values of 20 and 60 nm (Figure S1) [8]. Cross‐sectional EDS analysis further confirmed the layered Ni/SiO_*x*_/*n*‐Si structure and the spatial distribution of Si, O, and Ni across the electrode (Figure S2). AFM analysis confirmed the nanoscale uniformity of the Ni films, with low root‐mean‐square (rms) roughness values for both Ni thicknesses (Figure S3) [16, 17]. In addition, full XPS survey spectra confirmed the presence of Si, O, and Ni elements at the electrode surface, consistent with the expected MIS structure (Figure S4). These results are consistent with our previous studies on closely related Si/SiO_*x*_/metal‐based MIS photoelectrodes [9, 18]. Comparative evaluation revealed that photoanodes incorporating a 20 nm‐thick Ni layer exhibited superior performance for OER relative to those with a 60 nm‐thick Ni layer, as evidenced by cyclic voltammograms (CV) measurements (Figure S5). Specifically, the photoanode with the 20 nm‐thick Ni layer exhibited the highest photocurrent density owing to a reduced light absorption by thinner Ni and the lowest onset potential, indicating superior catalytic activity toward the OER. Consequently, the 20 nm thickness was adopted for all subsequent experiments.

**FIGURE 1:**
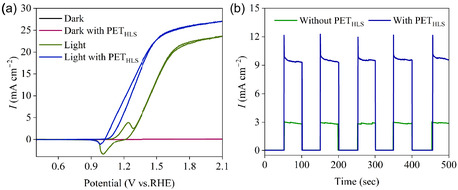
(a) Cyclic voltammograms (CVs) of the Ni/SiO_*x*_/*n*‐Si photoanode in 1 M KOH under dark and simulated one‐sun illumination, with and without PET_HLS_. (b) Corresponding current–time (*I–t*) curves recorded at 1.3 V versus RHE under chopped illumination.

PET_HLS_ containing terephthalic acid (TPA) and EG was prepared by alkaline hydrolysis of PET powder following the procedure described in the Supporting Information. The catalytic performance of the 20 nm‐thick Ni/SiO_*x*_/Si (denoted Ni/*n*‐Si hereafter) photoanode toward EG oxidation in diluted PET_HLS_ was evaluated in a two‐compartment PEC cell separated by a membrane under simulated solar illumination. To evaluate the role of Ni‐modified silicon photoanodes in directing photogenerated holes toward selective EG oxidation, the PEC performance of an Ni/*n*‐Si photoanode was preliminarily examined in 1 M KOH under dark and simulated solar illumination (Figure 1a). In the absence of PET_HLS_, a quasireversible redox feature centered at ∼1.24 V versus reversible hydrogen electrode (RHE) was observed, attributable to the Ni^3+^/Ni^2+^ redox couple associated with the NiOOH/Ni(OH)_2_ conversion in alkaline media [9, 19]. Upon addition of PET_HLS_, the photocurrent density increased markedly, accompanied by a shift in the onset potential reduced by at least 200 mV, indicating a thermodynamically more favorable PET_HLS_ oxidation relative to the OER [20]. Chopped‐light PEC chronoamperometric (*I–t*) measurements recorded at a constant applied potential further confirm the enhanced photocurrent response in the presence of PET_HLS_ (Figure 1b). The reproducible light on/off transients indicate efficient photogenerated charge separation and support the assignment of the increased anodic current to PET_HLS_ oxidation occurring at the Ni /*n*‐Si photoanode [5, 8].

**FIGURE 2:**
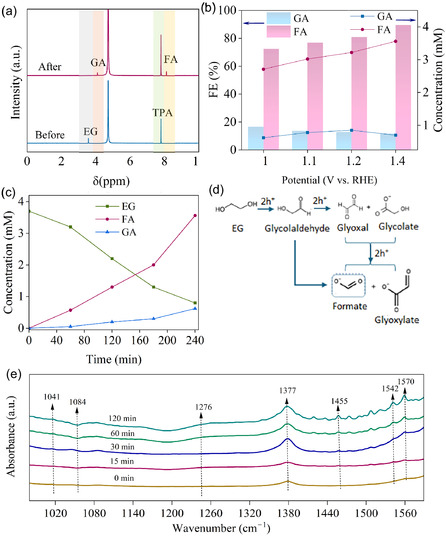
(a) ^1^H NMR spectra of PET_HLS_ before and after PEC oxidation at 1.2 V versus RHE for 240 min with the Ni/n‐Si photoanode. (b) Faradaic efficiency (FE) and concentrations of FA and GA at different applied potentials. (c) Concentrations of EG and its oxidation products during PEC oxidation of PET_HLS_ under simulated solar‐light illumination at an applied potential of 1.2 V versus RHE. (d) Proposed reaction pathway for the PEC oxidation of EG in alkaline solution. (e) In situ ATR‐FTIR spectra of PEC oxidation of PET_HLS_ at Ni/*n*‐Si (1.2 V vs. RHE from 0 to 120 min).

To identify the oxidation products of PET_HLS_, PEC experiments were conducted in a PET_HLS_ solution containing 3.8 mM EG. The electrolyte before and after electrolysis was analyzed by ^1^H NMR spectroscopy. An intense peak at 4.5–4.7 ppm was observed, corresponding to the residual water signal in both spectra. As illustrated in Figure 2a, the chemical shifts at 3.5 and 7.8 ppm correspond to EG and TPA in the starting PET_HLS_, respectively, indicating successful PET_HLS_ hydrolysis. Following PEC oxidation, EG in PET_HLS_ undergoes oxidation to form GA at 4.1 ppm and FA at 8.3 ppm, while TPA remains unreacted [7, 13, 21]. Consequently, PET derived EG is converted into the high added‐value products GA and FA. To maximize the economic potential of waste PET plastic upcycling, PET_HLS_ after electrolysis was separated and purified (Figure S6). Hence, the PEC oxidation of PET‐derived EG to value‐added FA and GA products enhances the profitability of PET reclamation, underscoring its practical applicability [3].

The reaction progress and product distribution during PET_HLS_ electrolysis were evaluated by high‐performance liquid chromatography (HPLC), with concentrations quantified using calibration curves of standard compounds. In comparison with photocatalytic and electrocatalytic approaches, the PEC technique enables faster PET_HLS_ oxidation under simulated solar‐light illumination at an applied bias of 1.0 V versus RHE (Figures S7−S10) [3, 22].

By comparing the Faradaic efficiencies (FE) at different applied potentials, FA is identified as the major product, accounting for ∼90% of the total FE over a broad potential range of 1.0–1.4 V versus RHE (Figures 2b and S11). This result is consistent with the accumulation of a high formate concentration in the electrolyte during the PEC process. This high selectivity highlights the specific role of the Ni/SiO_*x*_/*n*‐Si MIS architecture in PET‐derived EG oxidation. In previously reported PEC PET oxidation systems, Ni‐based cocatalysts were generally deposited on oxide semiconductor photoabsorbers to provide Ni^2+^/Ni^3+^ redox‐active sites for organic oxidation [11, 15]. In contrast, in the present Ni/SiO_*x*_/*n*‐Si photoanode, the thin Ni layer is integrated into the silicon‐based MIS architecture as a multifunctional catalytic contact [8, 9]. During PEC operation in alkaline electrolyte, the electrolyte facing Ni surface is oxidized to Ni^3+^/NiOOH like species, which act as active redox sites for EG oxidation. At the same time, the underlying Ni/SiO_*x*_/*n*‐Si junction facilitates photogenerated hole extraction from crystalline silicon and contributes to the stabilization of the Si photoabsorber under oxidative alkaline conditions. Therefore, the Ni layer simultaneously provides catalytic activity, interfacial charge transfer, and photoanode protection, which accounts for the high formate selectivity and stable PEC performance observed in this work.

HPLC analysis was further employed to monitor the time‐dependent conversion of EG on the Ni/*n*‐Si photoanode in a batch PEC reaction (Figures 2c and S12). Only a small amount of GA was detected during EG oxidation, suggesting that glycolaldehyde may be involved as a key transient intermediate rather than GA being the dominant intermediate [21, 23]. Zhou et al. reported that the reaction rates of glycolaldehyde, EG, and GA toward formate formation follow the decreasing order: glycolaldehyde > EG > GA, indicating that GA is unlikely to be the main intermediate in the conversion pathway toward formate [3]. Product analysis indicates that PET‐derived EG is converted through glycolaldehyde/glyoxal‐type C_2_ oxygenated intermediates at the photoanode surface. These intermediates may subsequently follow two possible oxidation pathways: oxidative C─C bond cleavage to produce formate, or further oxidation to glyoxal and glycolate, which may then undergo slower C─C bond cleavage to yield formate (Figure 2d) [7, 13, 23, 24]. Therefore, this pathway should be considered a plausible mechanistic interpretation consistent with the product distribution and previous EG oxidation studies.

Under illumination, the Ni/*n*‐Si photoanode generates electron–hole pairs, where photogenerated holes migrate to the photoanode/electrolyte interface while electrons are extracted through the external circuit toward the cathode. The PEC conversion of EG is therefore most reasonably attributed to a direct interfacial process driven by photogenerated holes at the Ni/*n*‐Si photoanode [9]. To further probe the reaction pathway, in situ attenuated total reflectance Fourier transform infrared (ATR‐FTIR) spectroscopy was employed to probe possible surface/interfacial intermediates formed during PEC oxidation of EG derived from PET depolymerization. As shown in Figure 2e, intense inverted bands at 1041 and 1084 cm^−1^ are assigned to C–O stretching vibrations of aldehyde functionalities in surface‐adsorbed glyoxal species, consistent with literature DFT predictions of preferential C–O coordination of C_2_ aldehyde intermediates on oxide surfaces [15, 24]. The appearance of a weak band at 1276 cm^−1^ indicates the formation of glyoxylate, reflecting progressive oxidation of glyoxal intermediates [24, 25]. A distinct band at 1377 cm^−1^, characteristic of symmetric COO^‒^ stretching in glycolate, suggests partial desorption of glyoxal followed by surface‐mediated rearrangement into glycolate via a Cannizzaro‐type pathway [23]. Additional bands at 1570, 1542, and 1455 cm^−1^ correspond to asymmetric and symmetric COO^−^ stretching vibrations of formate and glycolate species, consistent with the formation of formate and glycolate containing products during EG oxidation [14].

These spectroscopic observations are consistent with a possible mechanistic sequence involving initial alcohol deprotonation to surface alkoxide‐type species, followed by oxidation to glycolaldehyde/glyoxal‐type intermediates and further conversion to FA through C─C bond cleavage [23, 26, 27]. In related electrocatalytic systems, GA has been attributed to a Cannizzaro‐type transformation of desorbed glyoxal under alkaline conditions. The mechanistic parallels between electrocatalytic and present PEC systems suggest that glyoxal or glycolaldehyde‐type species may act as important C_2_ intermediates preceding formate formation [23]. Overall, these results support a PEC oxidation pathway in which interfacial hole transfer at the Ni/SiO_*x*_/*n*‐Si photoanode promotes the conversion of PET‐derived EG into FA and GA, although the exact elementary steps of C─C bond scission remain to be directly resolved [28, 29].

**FIGURE 3:**
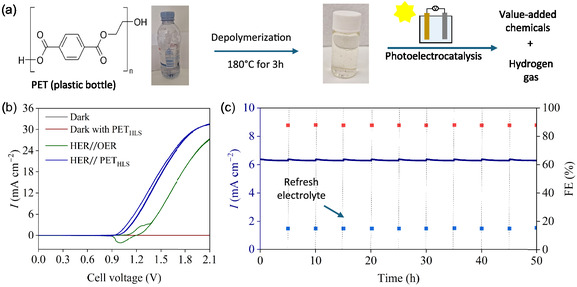
(a) Schematic illustration for the solar‐driven formate production from waste PET plastic bottle. (b) CV curves obtained for the two‐compartment PEC cell in 1 M KOH under different conditions: in the dark without and with PET_HLS_; under simulated solar illumination for HER//OER and HER//PET_HLS_ configurations. (c) Chronoamperometric curve (dark blue line) for the oxidation of PET_HLS_ at an applied cell voltage of 1.1 V under simulated solar illumination and corresponding FE for formate (red squares) and glycolate (blue squares).

Considering the favorable catalytic performance toward PET_HLS_ oxidation, the catalytic system was integrated into a solar simulator‐driven PEC device operated in an anion‐exchange membrane‐separated, two‐electrode system under alkaline conditions (Figures 3a and S13). To compare device performance, CV measurements were carried out for both water splitting (HER//OER) and PET_HLS_ oxidation (HER//PET_HLS_) within the same PEC setup (Figure 3b). Nearly parallel polarization curves were observed for water splitting and PET oxidation, with a less positive shift in cell voltage (approximately 200 mV) required to reach the same photocurrent density in the presence of PET‐derived reactants, which in turn increases the cathodic energy efficiency [15, 28].

To further evaluate the practical feasibility of the coupled system, the long‐term stability of the Ni/*n*‐Si photoanode for the solar‐driven oxidation of PET_HLS_ was also examined at an applied cell voltage of 1.1 V. FA and GA were identified as the main anodic products (Figures 3c and S14). Notably, the FEs for FA and GA remained stable over 50 h of continuous operation, demonstrating excellent operational durability. Overall, this PEC tandem system achieved a combined FA and GA production rate corresponding to a formate productivity of 6240 μmol cm^−2^ h^−1^, outperforming most previous reports (Table S1). The corresponding mass‐normalized formate productivity was calculated to be 3.51 × 10^5^ μmol mg_Ni_^−1^ h^−1^. At the applied operating voltage, the system exhibited high selectivity and stable product yields, highlighting its improved economic feasibility and efficient solar energy utilization through the tandem integration of PET_HLS_ oxidation and the HER.

**FIGURE 4:**
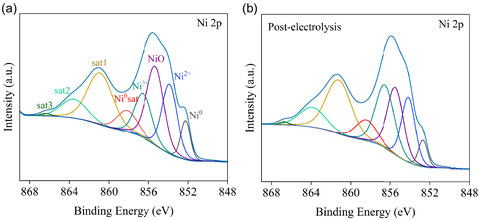
High‐resolution XPS spectra of Ni 2p obtained for the Ni/*n*‐Si photoanode (a) before and (b) after long‐term stability test at 1.1 V under simulated solar illumination.

On the other hand, XPS was employed to investigate the surface chemical states of Ni and O before and after PEC oxidation of PET_HLS_ at the Ni/*n*‐Si photoanode in a two‐electrode cell (Figures 4 and S15). The Ni 2p spectra exhibit the characteristic spin–orbit doublet of Ni 2p_3/2_ and Ni 2p_1/2_ accompanied by intense shake‐up satellite features, confirming the predominance of oxidized Ni species at the surface. Detailed deconvolution of the Ni 2p_3/2_ region reveals a minor metallic Ni^0^ contribution at 852.7 eV, indicating that the freshly prepared electrode retains a metallic Ni core. The oxidized components are centered at 854.2 eV, assigned to Ni^2+^ in NiO, and at higher binding energies (∼855.5–858.4 eV), corresponding to Ni^2+^ species in Ni(OH)_2_/NiOOH environments. A component at ∼856.5 eV is consistent with Ni^3+^ species, characteristic of NiOOH formed under anodic polarization [7, 30, 31]. The spectrum also shows pronounced satellite peaks at ∼861.2, 863.8, and 866.7 eV, which arise from multiple splitting and charge transfer processes typical of Ni^2+^ compounds, further confirming the dominance of oxidized nickel at the outermost surface. After prolonged electrolysis, the relative contribution of the Ni^3+^ component increases while the metallic Ni^0^ signal becomes strongly attenuated, indicating progressive surface oxidation and the formation of a NiOOH‐rich oxyhydroxide layer under photoanodic operating conditions [9, 31]. This evolution suggests an increase in the Ni^3+^/Ni–O ratio after electrolysis, consistent with the electrochemical activation of the nickel surface.

Consistently, the O 1s envelope can be deconvoluted into a lower binding energy component at ∼530.8–531.0 eV attributed to lattice O^2^^−^ in Ni─O bonds, and a dominant component centered at ∼531.5–532.0 eV associated with hydroxyl species (Ni–OH) and/or oxyhydroxide environments. Another lower binding‐energy contribution at ca. 529.0 eV is visible and assigned to adsorbed water or surface oxygen‐containing species. After the long‐term stability test under PEC operation, an increase in the relative intensity of the Ni–OH contribution is observed in the O 1s spectrum, indicating enhanced formation of hydroxylated NiOOH‐type surface species during electrolysis. This observation is consistent with the increase of Ni^3+^ detected in the Ni 2p spectra. Collectively, the Ni 2p and O 1s analyses demonstrate that although the initial material contains a metallic Ni core covered by a native oxide layer, anodic PEC conditions promote surface oxidation and the formation of a Ni^2+^/Ni^3+^ containing NiO_*x*_(OH)_*y*_ shell enriched in Ni^3+^ and Ni–OH species during PET_HLS_ oxidation, confirming the chemical robustness of the Ni‐based catalytic interface under operational conditions [19, 20]. In addition, top‐view SEM images recorded before and after PEC oxidation show that the Ni/*n*‐Si photoanode retains a homogeneous and continuous nanoscale surface morphology, with no visible cracks, delamination, pinholes, or large aggregates (Figure S16). Overall, the SEM and XPS analyses confirm the preservation of the surface morphology and chemical robustness of the Ni‐based catalytic layer after prolonged PEC operation.

These results demonstrate the excellent surface stability of the Ni‐based active layer under PEC oxidation of PET_HLS_, further confirming the suitability of the Ni/*n*‐Si photoanode for sustained PEC oxidation reactions. Therefore, this work presents a solar‐driven chemical conversion strategy with a reduced carbon footprint, holding promise for the integration of intermittent renewable energy in future practical applications. In addition, the present PEC approach offers attractive commercial advantages compared with conventional waste photoreforming processes, which typically yield complex mixtures of poorly utilizable products [32, 33]. By contrast, our system enables the selective formation of value‐added chemicals with substantially enhanced production rates and operational flexibility. Consequently, this strategy represents a significant advance over traditional waste photo‐reforming, approaching key performance metrics required for commercially viable plastic waste utilization [34, 35].

## 3 Conclusion

In summary, we demonstrate a solar‐driven PEC strategy for selective C─C bond valorization of bottle‐derived EG using a stable Ni/SiO_*x*_/*n*‐Si MIS photoanode. Under simulated solar irradiation, the catalytic photoanode promotes highly selective oxidation of PET_HLS_‐derived EG to FA, delivering sustained activity and long‐term operational stability. Mechanistic insights were obtained using in situ ATR‐FTIR spectroscopy, which enables identification of surface‐bound intermediates involved in the PEC oxidation pathway leading to C─C bond cleavage. The two‐compartment PEC tandem cell operated in a membrane‐separated configuration under simulated solar irradiation, achieves a combined FA and GA production rate corresponding to a formate productivity of 6240 μmol cm^−2^ h^−1^. These findings establish silicon‐based photoanodes as effective platforms for controlled plastic‐derived carbon transformations and provide a viable pathway toward solar‐assisted PET waste upcycling.

## Funding

This study was supported by the European Union’s Horizon Europe Programme (Grants HORIZON‐MSCA‐2024‐PF‐01, project SOLAR‐CATMOF).

## Conflicts of Interest

The authors declare no conflicts of interest.

## Supporting information

Supplementary Material

## Data Availability

The data that support the findings of this study are available from the corresponding author upon reasonable request.
