# Supplementary material for: Photoelectrochemical Valorization of Plastic Waste Using Catalytic Silicon Photoanodes
Source: ChemSusChem. 2026 Jun 30;19(13):e70852. doi: 10.1002/cssc.70852 (PMC13318168; doi:10.1002/cssc.70852)
Supplement: Supplementary file 1 — Supplementary Material [file CSSC-19-e70852-s001.pdf]

Supporting Information

Photoelectrochemical Valorisation of Plastic Waste Using Catalytic Silicon Photoanodes

Manel Machreki, [a] Marielle Blot, [a] Gabriel Loget, [b] Patrick Garrigue, [b] Isabelle Soutrel,[c] and Bruno Fabre[*a]

[a] Univ Rennes, CNRS, ISCR (Institut des Sciences Chimiques de Rennes)-UMR6226, F-35000 Rennes, France

[b] University of Bordeaux, Bordeaux INP, ISM, UMR CNRS 5255, Pessac 33607, France

[c] Univ Rennes, Ecole Nationale Supérieure de Chimie de Rennes, CNRS, ISCR-UMR 6226, F-35000 Rennes, France

Corresponding author: E-mail: bruno.fabre@univ-rennes.fr

1. Experimental Details

1.1. Chemicals and materials

Sodium sulfite (Na_2SO_3, >99%), tert-butyl alcohol, ethylene glycol ($HOCH_2CH_2OH$, 99.7%), formic acid ((FA), HCOOH, 99%), glycolic acid ((GA), 99%), sulfuric acid (H_2SO_4, 99.7%)), and potassium hydroxide (KOH, 99%) were purchased from Sigma-Aldrich. Deuterated water (D_2O) was purchased from Merck. All other chemical reagents were of analytical grade and used as received without further purification. The chemicals used for cleaning and etching the Si wafers were sulfuric acid (H_2SO_4 96%, Very Large Scale Integration (VLSI) grade, Selectipur) and hydrogen peroxide (H_2O_2 30%, VLSI grade, Sigma-Aldrich), purchased from VWR and Sigma-Aldrich, respectively, as well as acetone (electronic grade MOS, Erbatron by Carlo Erba Reagents) and anhydrous ethanol (RSE electronic grade, Erbatron by Carlo Erba Reagents). All electrolyte solutions were prepared with ultrapure water (18.2 MΩ·cm resistivity, Purelab Flex 2 from Veolia Water STI).

1.2. Preparation of SiO_x/n-Si

All Teflon vials and tweezers used for silicon cleaning were pre-decontaminated in a 3:1 (v/v) mixture of concentrated H_2SO_4 and 30% H_2O_2 at 105 °C for 30 min, followed by thorough rinsing with ultrapure water. *Caution*: Concentrated aqueous H_2SO_4/ H_2O_2 (piranha solution) is highly hazardous, particularly upon contact with organic materials, and must be handled with extreme care. The *n*-type silicon wafers (phosphorus-doped, resistivity 0.3–0.7 Ω·cm, thickness 475–525 µm, orientation (100)) were purchased from Electronics. All Si substrates were

degreased by sequential sonication in acetone, ethanol, and ultrapure water for 10 min each. The surfaces were subsequently decontaminated and oxidized in piranha solution at 105 °C for 30 min, rinsed extensively with ultrapure water, and dried under an argon flow.

1.3. Preparation of Ni/SiO$_x$/n-Si

Ni thin films (20 and 60 nm thicknesses) were deposited on SiO$_x$/*n*-Si by sputtering with a Leica EM ACE600 coating system (Ni target purity: 99.8%, Leica) technique. The method and characterizations of the films were described in our previous works [1, 2].

1.4. Depolymerization of PET

Model PET was employed as the plastic waste. The hydrolysis is carried out for the depolymerization of PET. In the beginning, PET plastic bottles were initially cut into small pieces and pulverized into PET powder using a household blender and then 3 g of PET plastic powder was washed with ethanol and deionized water. After that, the clean powder that had been dried was added to 30 mL of 1 M KOH solution and separately transferred to several Teflon-lined autoclaves, which were sealed and maintained at 180°C for 3 h. After cooling to room temperature, the resulting solution containing ethylene glycol (EG) and terephthalate was used as the electrolyte for PEC tests and analyses.

1.5. Instrumentation

X-ray photoelectron spectroscopy (XPS) data were collected using a NEXSA G2 (Thermo Fisher Scientific) spectrometer equipped with an Al Kα X-ray source operating at 1486.6 eV and a spot size of 200 μm^2. Survey spectra were acquired with a pass energy of 200 eV and a step size of 1 eV, while high-resolution spectra were recorded using a pass energy of 50 eV and a step size of 0.1 eV. All binding energies were referenced to the C 1s peak at 284.8 eV. Core-level spectra of Ni 2p and O 1s were peak-fitted using CasaXPS software (version 2.3.18), employing either a Tougaard or Shirley background for spectral analysis.

The analytical photoelectrochemical measurements (cyclic voltammograms CVs and stability test) were performed at room temperature in a three-electrode configuration on a BioLogic SP-200 single-channel electrochemical workstation. An alkaline solution (1.0 M KOH, pH = 14 ± 0.1) was used as the electrolytic solution. The light was provided by a solar simulator with a fluence of 100 mW cm^{-2} (LS0106, LOT Quantum Design) equipped with an AM 1.5G filter. A three-electrode system was used during the PEC experiments, where the Ni/*n*-Si photoanode served as the working electrode (geometric area ~0.15–0.20 cm^2), the

Hg/HgO electrode as the reference electrode, and a graphite plate as the counter electrode. All potentials measured vs. Hg/HgO were converted versus the reversible hydrogen electrode (RHE) according to the equation given below:

$$E_{RHE} = E_{Hg/HgO} + 0.059\ pH + E^0_{\ Hg/HgO} \text{ with } E^0_{\ Hg/HgO} = 0.098V \quad (1)$$

where $E_{Hg/HgO}$ is the applied potential versus the used reference electrode.

PEC oxidation of PET was also conducted in a two-compartment PEC cell comprising photoanodic and cathodic compartments separated by an anion-exchange membrane (FM-FAS-50, QuinTech). The photo-response of Ni/*n*-Si photoanode was studied by measuring photocurrent densities with and without the targeted molecule. After the chronoamperometric experiments at fixed potential, 0.5 mL aliquot was collected and neutralized with 0.5 mL of 0.05 M H_2SO_4 solution to identify and quantify the electrogenerated products.

Following electrolysis, the electrolytic solution was collected and subjected to nuclear magnetic resonance (NMR) spectroscopy for analysis (400 MHz Bruker Ascend). For the ^{1}H NMR analyses, 500 μL of electrolyte was combined with 100 μL of D_2O.

Additionally, for each high-performance liquid chromatography (HPLC) measurement (Agilent 1200: VWD detector UV 210 nm at 40°C), 100 μL of the electrolyte solution containing products was diluted to 1 mL with diluted H_2SO_4 solution and filtered to separate terephthalic acid (TPA) from the solution, and then 20 μL of the as-prepared sample was injected into a BioRad Aminex 87H column. A 2.5 mM H_2SO_4 solution was used as the mobile phase with a constant flow rate of 0.5 mL/min. The product categories were identified by comparing the retention times of the elution peaks with the individual standard sample solutions. The concentrations of the electrolysis products were calculated from the calibration curves made by measuring standard solutions with different concentrations. HPLC equipped with ultraviolet (UV) and refractive index detector was used for the calibration and identification of EG.

In situ attenuated total reflectance Fourier-transform infrared (ATR-FTIR) measurements were conducted to monitor the reaction intermediates formed at the photoelectrode–electrolyte interface during electrolysis. Spectra were recorded using an FTIR spectrometer (Vertex 70 from Brüker Optics) equipped with an ATR accessory. Spectra were collected in the range of 4000–800 cm^{-1} with a resolution of 4 cm^{-1}, averaging 32–64 scans per spectrum. Difference spectra were generated by subtracting the background spectrum, allowing identification of

adsorbed intermediates and reaction products. All spectra were processed using baseline correction and, where appropriate, normalization.

2. Calculations of the Conversion Efficiency, Yield Rate and Faradaic Efficiency:

The Faradaic efficiencies (FE) to produce GA and FA were calculated using the following equation:

$$\mathrm{FE} = \left(\frac{n \times z \times F}{Q}\right) \times 100 \tag{2}$$

where *n* is the amount of GA or FA produced (in mole), *z* is the number of electrons mole required to produce one mole of GA or FA, *Q* is the total charge passed (in C), and *F* is the Faraday constant (96485 C mol^{-1}).

The following equations were used to calculate the selectivity and the yield of product (*P*):

$$Yield(\%) = \left[\frac{C_P}{C_0}\right] \times 100 \tag{3}$$

where C_0, and C_P are the initial concentration of alcohol and the reactant concentration after time *t*, respectively.

The production rate of the product (*P*) was calculated according to:

$$\text{Formate productivity}\ (\text{mmol cm}^{-2}\ \text{h}^{-1}) = \frac{\text{amount of produced formate (mmol)}}{\text{area of photoanode (cm}^{2}) \times \text{reaction time (h)}} \tag{4}$$

3. Additional Figures and Table

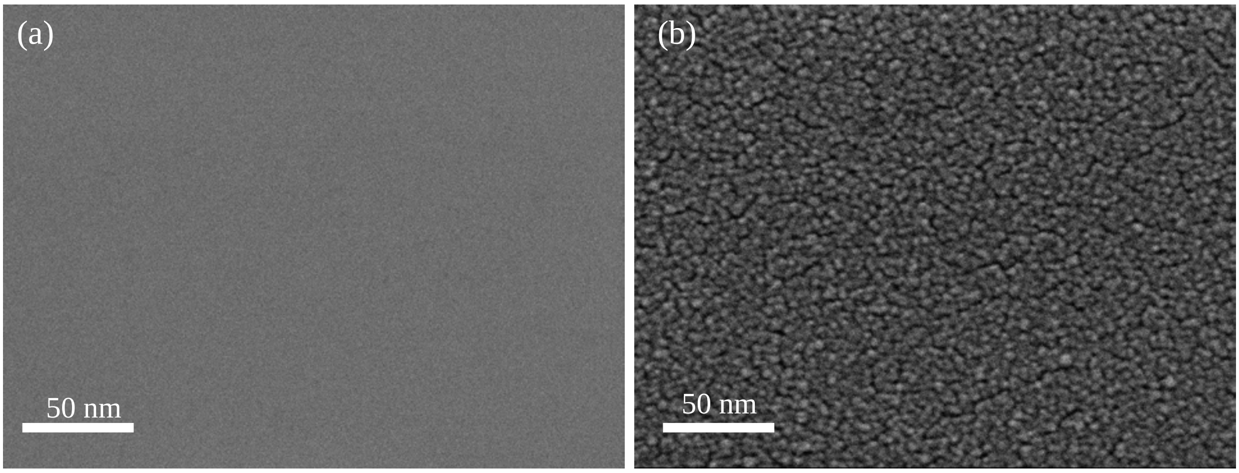

Fig. S1. Top-view SEM pictures of Ni/SiO_x/*n*-Si photoanodes with (a) 20- and (b) 60 nm-thick Ni layers.

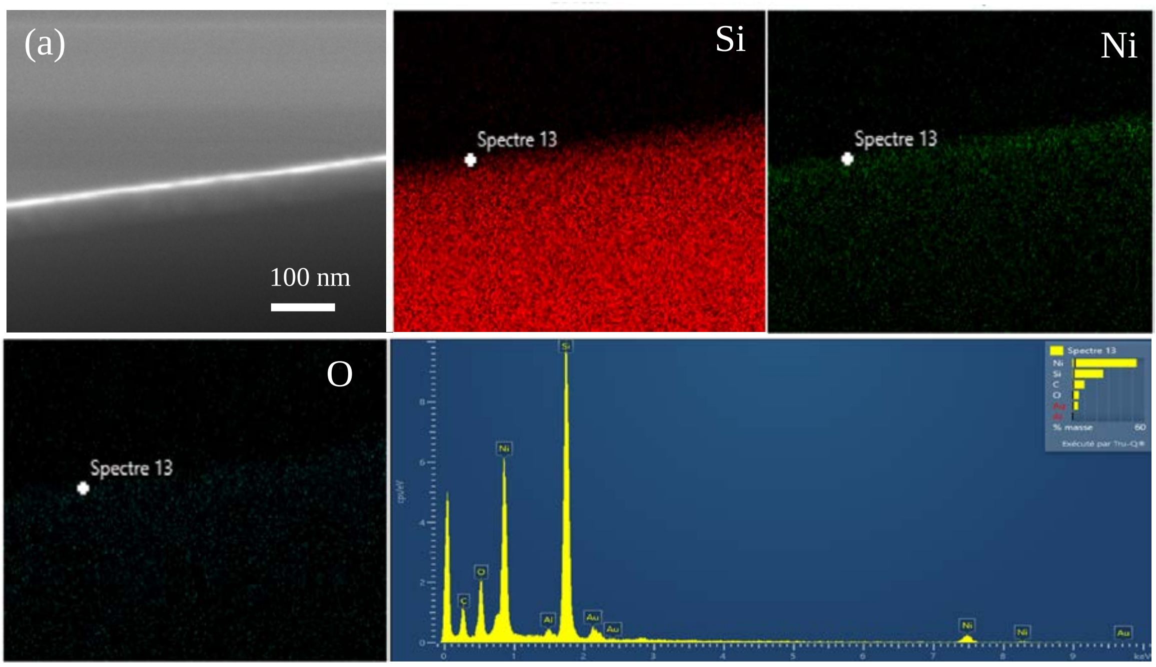

Fig. S2. Cross-section SEM image, corresponding EDS mapping for Si, Ni and O, and EDS spectrum for the 60 nm-thick Ni/SiO_x/*n*-Si photoanode.

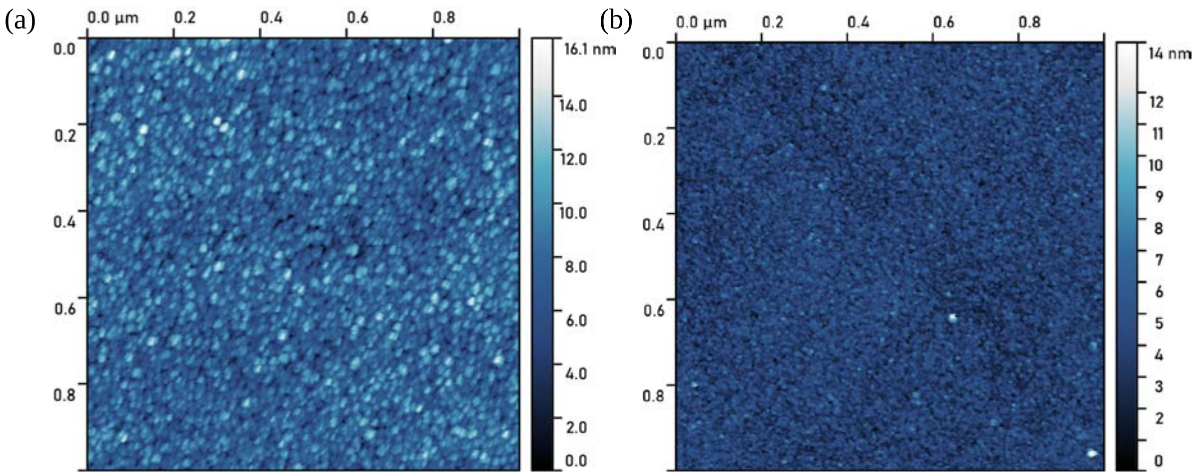

Fig. S3. AFM images of Ni/SiO_x/*n*-Si photoanodes with (a) 20- and (b) 60 nm-thick Ni layers. The 20 nm- and 60 nm-thick Ni films showed a low root-mean-square (rms) roughness of 1.2 nm and 1.9 nm, respectively.

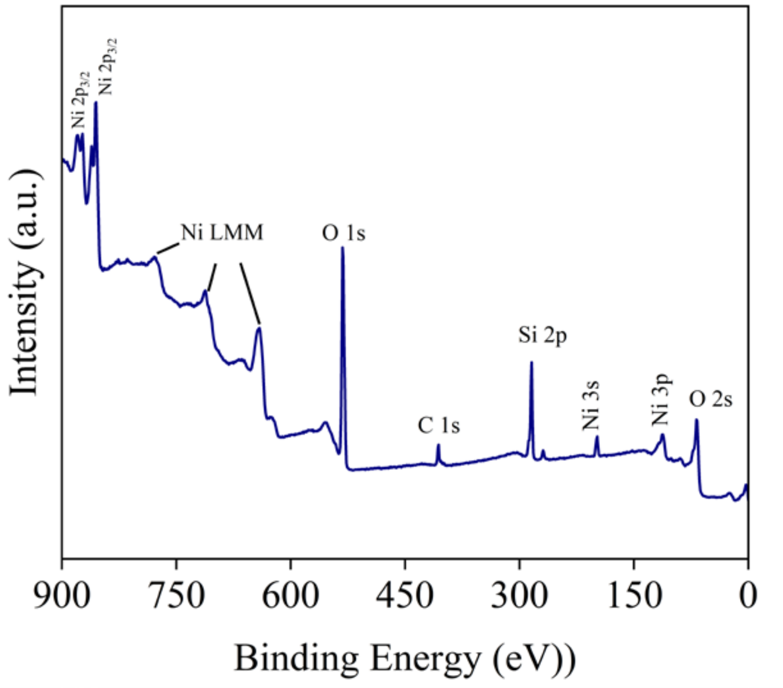

Fig. S4. Survey XPS spectrum for 20 nm-thick Ni/SiO_x/*n*-Si photoanode.

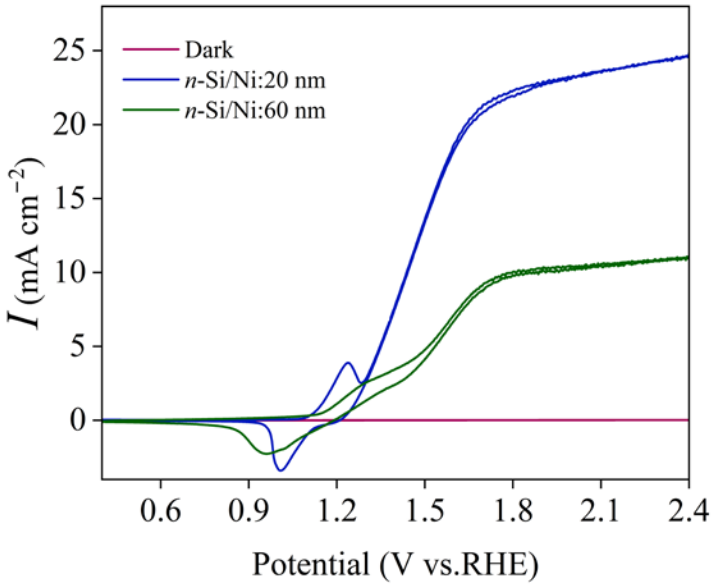

Fig. S5. Cyclic voltammograms (CVs) of Ni/SiO_x/*n*-Si photoanodes with 20- and 60 nm-thick Ni layers in 1 M KOH under dark and illuminated conditions.

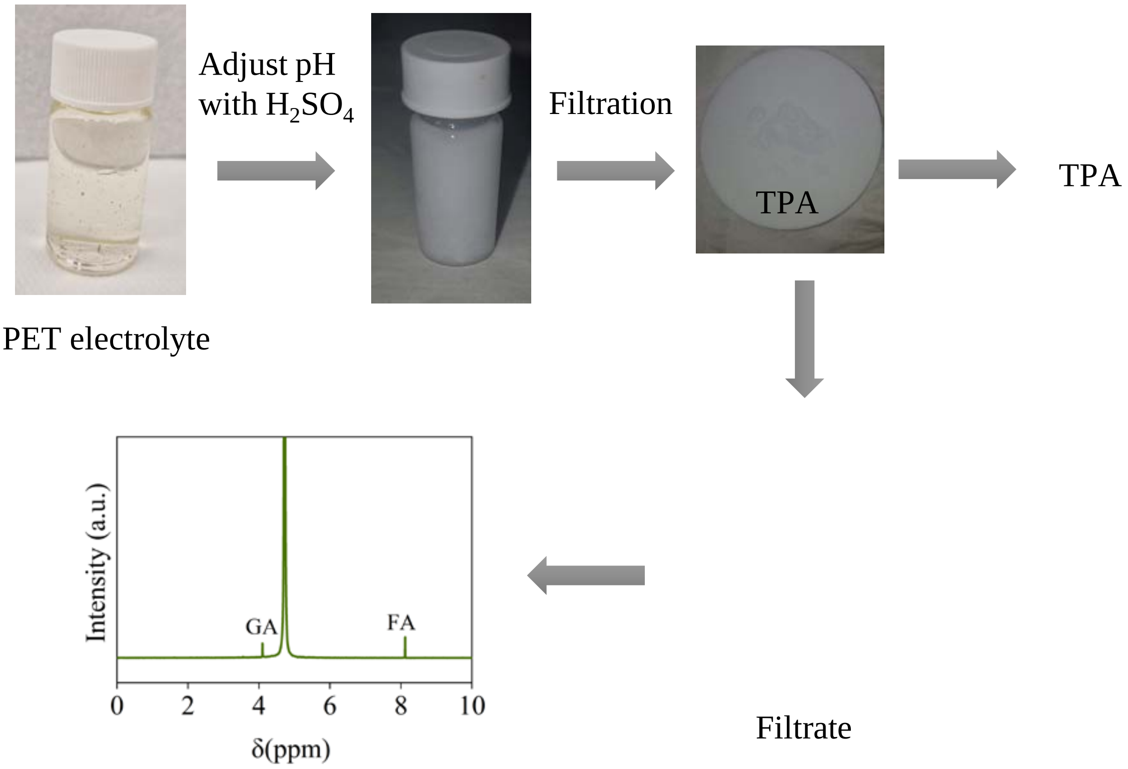

Fig. S6. Photographs showing the different steps of products separation from PET_{HLS} electrolyte after photoelectrolysis. The curve corresponds to the ^{1}H NMR spectrum of PET_{HLS} after PEC oxidation (filtrate solution) at 1.2 V vs. RHE for 240 min using the Ni/*n*-Si photoanode.

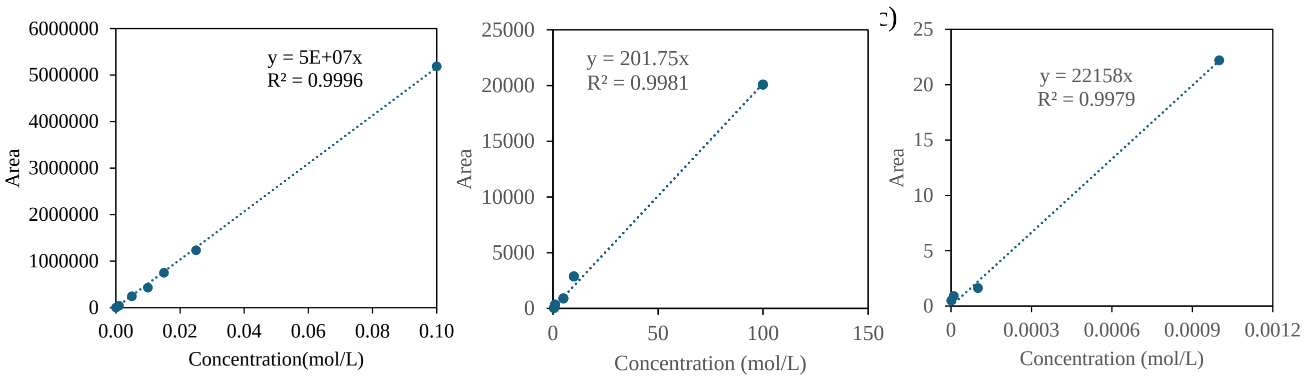

Fig. S7. HPLC calibration curves of (a) EG, (b) FA and (c) GA standard samples.

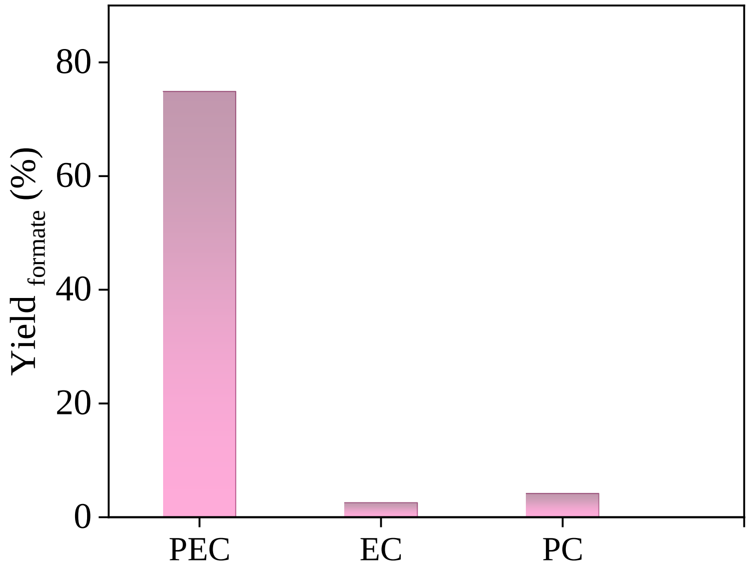

Fig. S8. Performance comparison of photocatalytic (PC, under simulated solar-light illumination), electrochemical (EC, at an applied potential of 1.0 V vs RHE in the dark), and photoelectrochemical (PEC, under simulated solar-light illumination at an applied potential of 1.0 V vs RHE) methods for producing formate from PET_{HLS} oxidation at the Ni/*n*-Si (photo)anode.

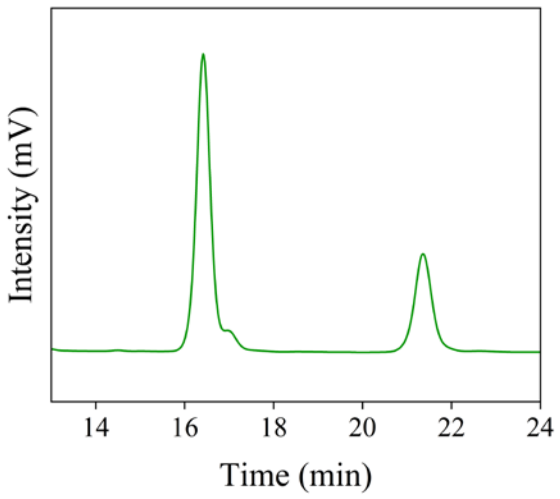

Fig. S9. HPLC chromatogram of EG after PET hydrolysate. The intense peak at ~16.4 min corresponds to EG.

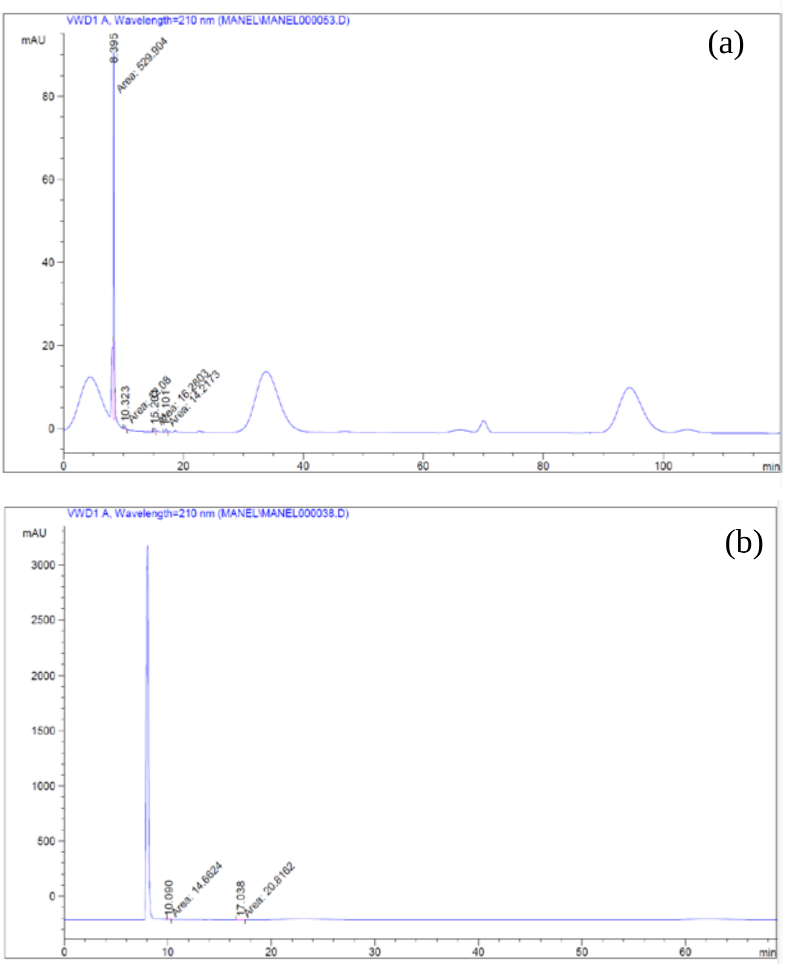

Fig. S10. HPLC chromatograms of PET_{HLS} oxidation products obtained on the Ni/*n*-Si photoanode after 240 min of (a) electrochemical (EC, at an applied potential of 1.0 V vs RHE in the dark) and (b) photocatalytic (PC, under simulated solar-light illumination). The intense peak at ~8.5 min corresponds to terephthalic acid and the peak at ~17.1 min corresponds to FA.

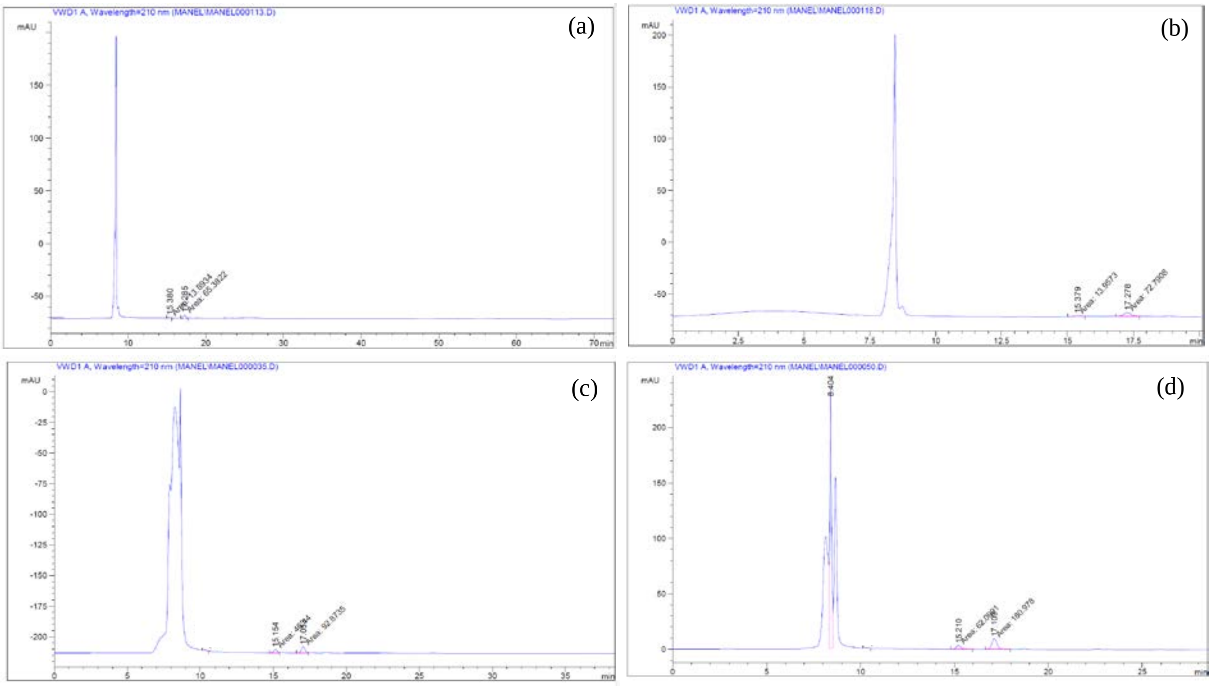

Fig. S11. HPLC chromatograms of PEC PET_{HLS} oxidation products obtained after 240 min at (a) 1.0, (b) 1.2, (c) 1.3 and (d) 1.4 V vs RHE. The intense peak at ~8.5 min corresponds to terephthalic acid, while peaks at 15.2-15.4 and 17.1-17.3 min correspond to GA and FA, respectively.

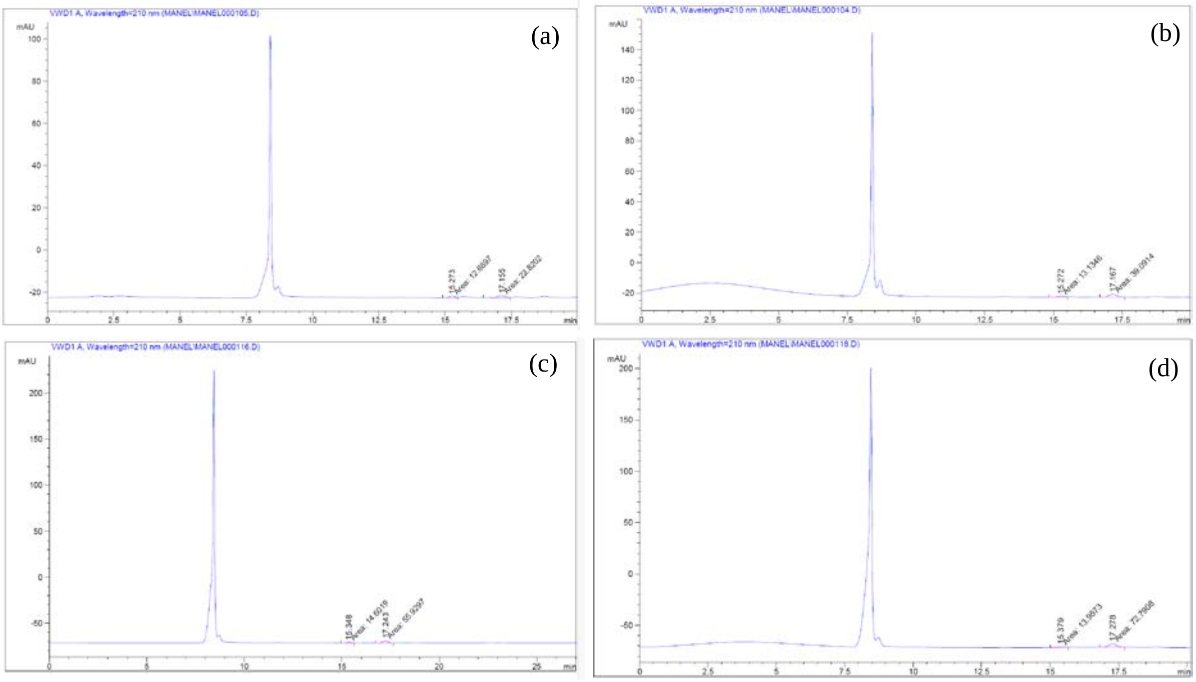

Fig. S12. HPLC chromatograms of PEC PET_{HLS} oxidation products obtained after (a) 60, (b) 120, (c) 180 and (d) 240 min at 1.2 V vs RHE. The intense peak at ~8.5 min corresponds to terephthalic acid, while peaks at 15.2-15.4 and 17.1-17.3 min correspond to GA and FA, respectively.

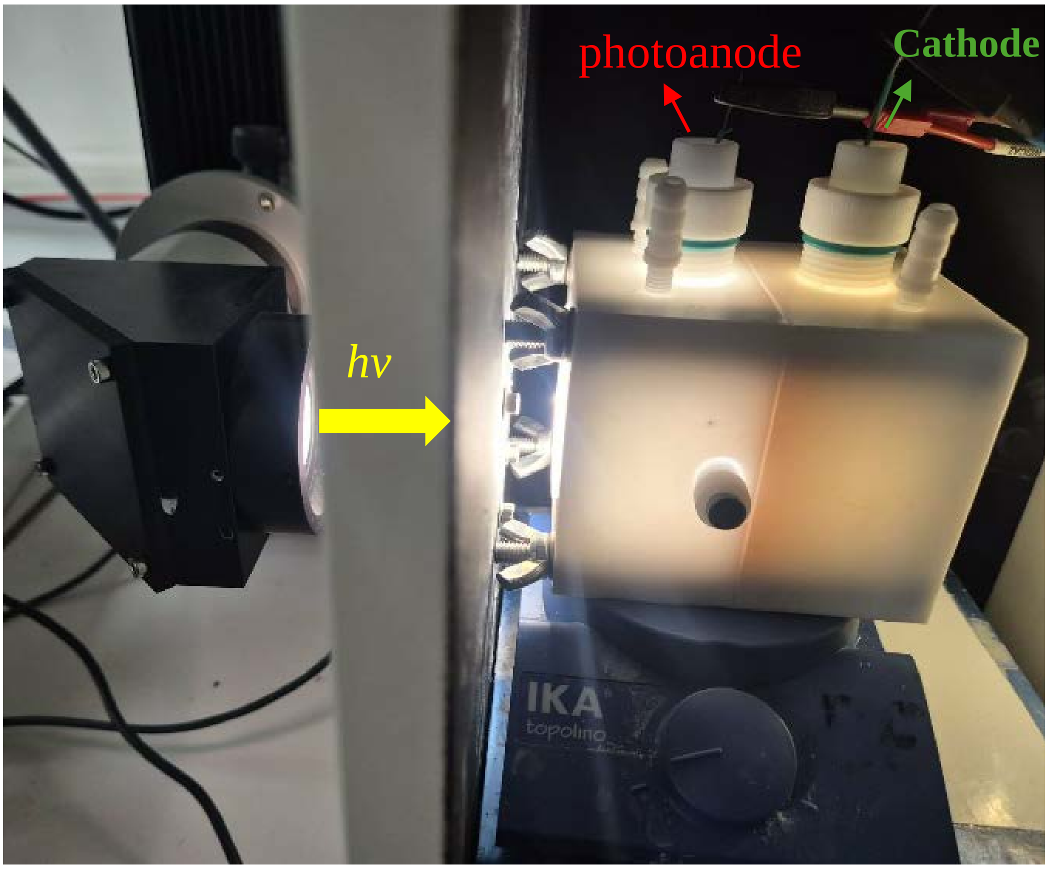

Fig. S13. Photograph of the two-electrode cell used to investigate the cathodic hydrogen evolution reaction (HER) coupled with anodic PET hydrolysate oxidation reaction (EGOR), employing a Pt electrode as the cathode and an Ni/*n*-Si electrode as the photoanode. The two-electrode electrolyzer consisted of 1 M KOH in the cathodic compartment and PET hydrolysate in the anodic compartment, separated by an anion exchange membrane.

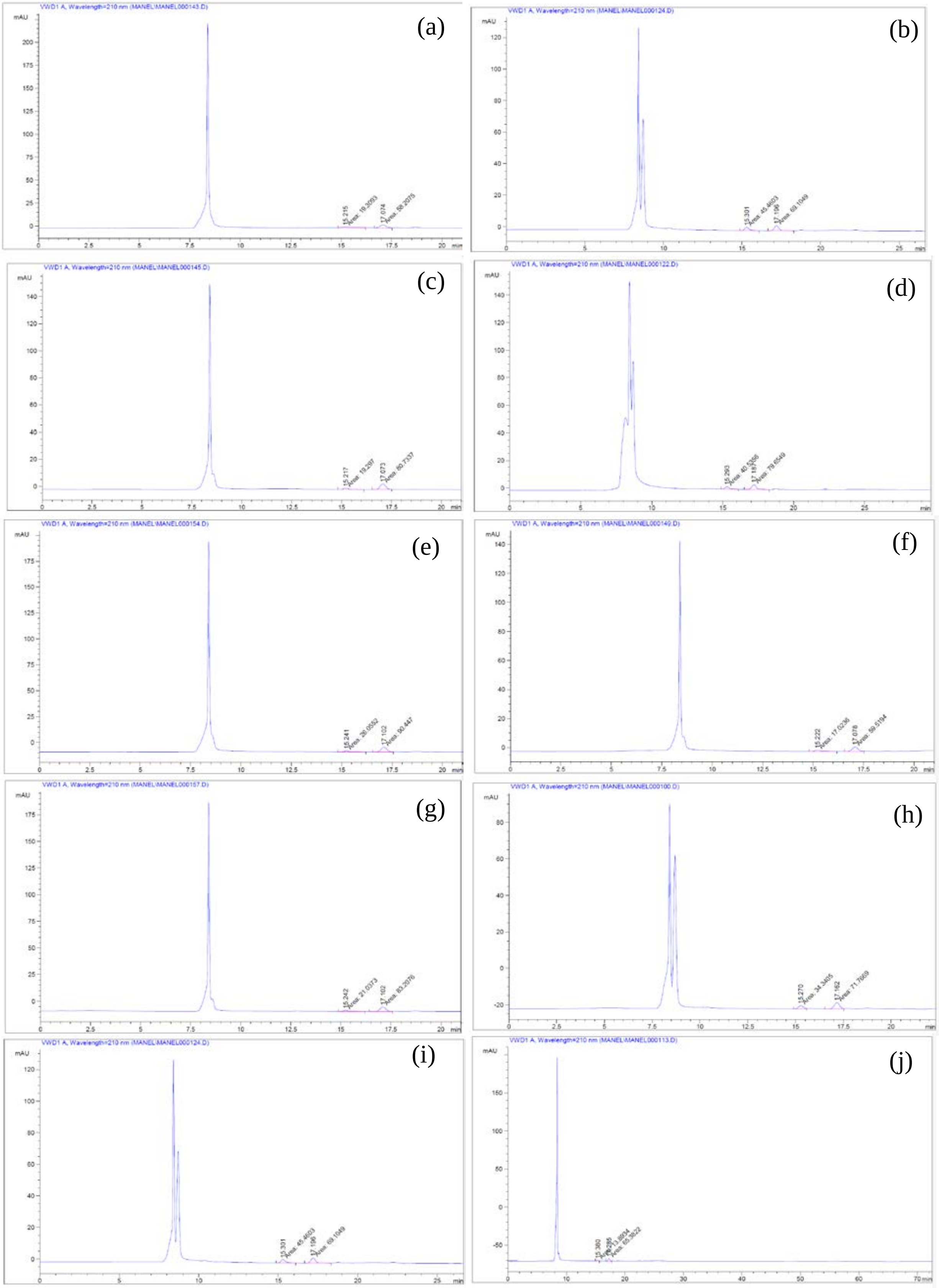

Fig. S14. HPLC chromatograms of PET_{HLS} oxidation products produced at an applied cell voltage of 1.1 V under simulated solar illumination at different electrolysis times: (a) 5, (b) 10, (c) 15, (d) 20, (e) 25, (f) 30, (g) 35, (h) 40, (i) 45 and (j) 50 hours.

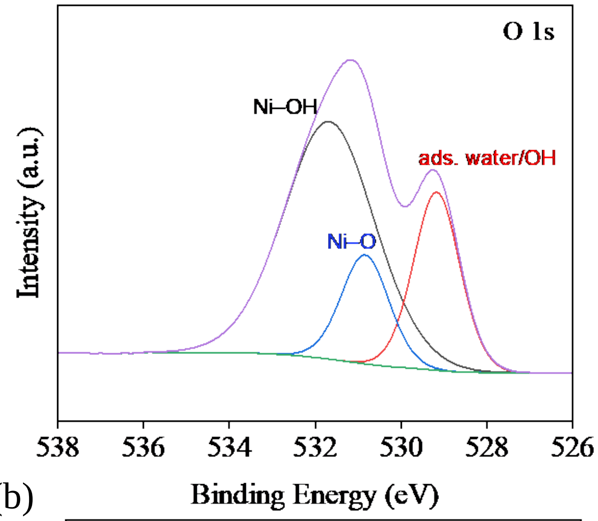

(b)

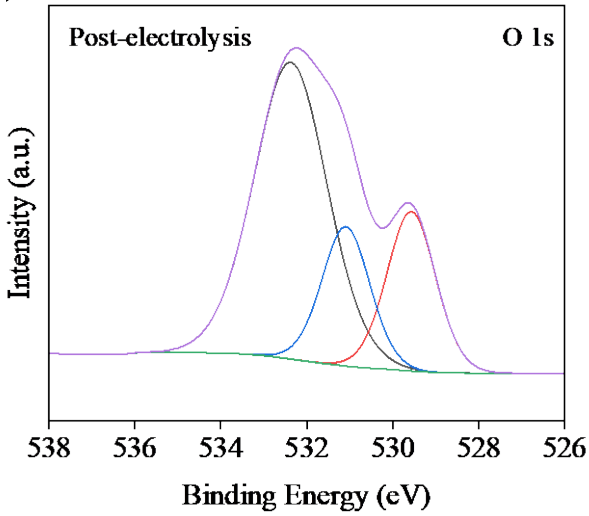

Fig. S15. High-resolution XPS spectra of O 1s obtained for the Ni/*n*-Si photoanode (a) before and (b) after long-term electrolysis at 1.1 V under simulated solar illumination.

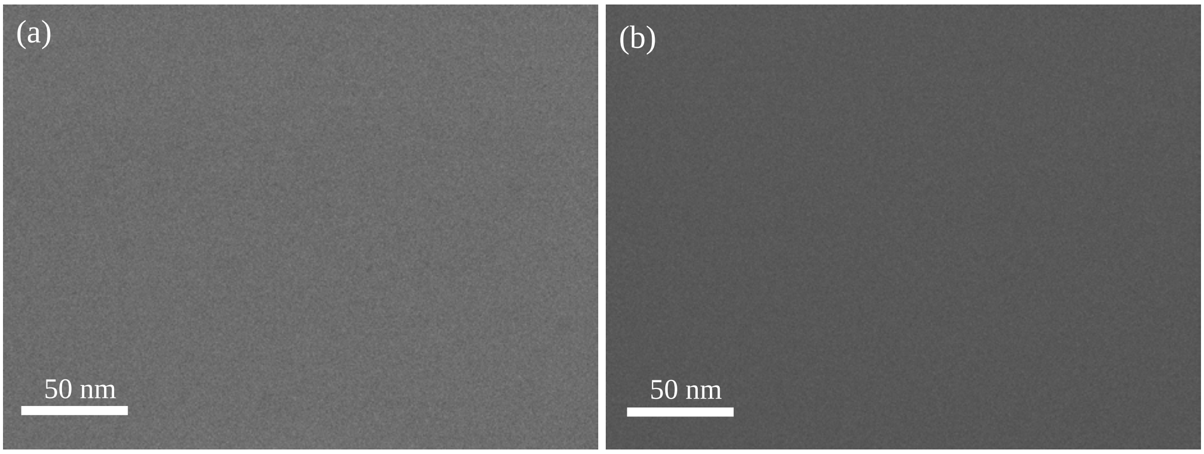

Fig. S16. Top-view SEM images of 20 nm-thick Ni/SiO_x/*n*-Si photoanodes (a) before and (b) after long-term electrolysis.

Table S1. Comparison among state-of-the-art PEC tandem cells for the formate production from solar-driven oxidation of PET hydrolysate coupled to HER (HER//PET oxidation).

**Photoelectrochemical tandem device**	**Formate production rate (*μ*mol cm^{-2} h^{-1})**	**Formate FE (%)**	**Stability (h)**	**References**
RuO_x|Cu_2O||Mo: $BiVO_4$|NiCo-LDH	4.8	40	8.3	[3]
BNN@NF|perovskite|BNN@NF	42	–	~10 h	[4]
TiO_2||Pt	16.6	85	3	[5]
Ni/*n*-Si ||Pt	6240	87	50	This work

4. References

[1] G. Loget, C. Mériadec, V. Dorcet, B. Fabre, A. Vacher, S. Fryars, and S. Ababou-Girard, "Tailoring the photoelectrochemistry of catalytic metal-insulator-semiconductor (MIS) photoanodes by a dissolution method," *Nature Communications* **10** (2019): 3522, https://doi.org/10.1038/s41467-019-11432-1.

[2] J. Dabboussi, R. Abdallah, L. Santinacci, S. Zanna, A. Vacher, V. Dorcet, S. Fryars, D. Flonera, and G. Loget, "Solar-assisted urea oxidation at silicon photoanodes promoted by an amorphous and optically adaptive Ni–Mo–O catalytic layer," *Journal of Materials Chemistry A* **10** (2022): 19769–19777, https://doi.org/10.1039/D2TA01567H.

[3] F. Kang, Q. Wang, D. Du, L. Wu, D. W. F. Cheung, J. Luo, Photoelectrochemical Ethylene Glycol Oxidation Coupled with Hydrogen Generation Using Metal Oxide Photoelectrodes, *Angew. Chem. Int. Ed.* **64** (2025): e202417648. https://doi.org/10.1002/anie.202417648.

[4] Y. Zhu, Y. Li, A. Zhang, Q. Chang, F. Liu, Z. Chen, X. Wang, R. Zhang, W. A. Daoud, Perovskite-Driven Solar Reforming of PET Waste and Concurrent Hydrogen Production, *Nano Energy* **146** (2025): 111517. https://doi.org/10.1016/j.nanoen.2025.111517.

[5] V. T. Ngo, K. Gulati, C. S. Law, N. Q. H. Tran, J. Lin, D. L. Stachura, A. D. Abell, H. Zhang, A. Santos, Titania Nanopores as Photoelectrocatalysts for Coupling Hydrogen

Production with Plastic Reformation, *Adv. Sci.* **12** (2025): e09287. https://doi.org/10.1002/advs.202509287.
